# Sox9-Regulated miRNA-574-3p Inhibits Chondrogenic Differentiation of Mesenchymal Stem Cells

**DOI:** 10.1371/journal.pone.0062582

**Published:** 2013-04-23

**Authors:** David Guérit, Didier Philipot, Paul Chuchana, Karine Toupet, Jean-Marc Brondello, Marc Mathieu, Christian Jorgensen, Danièle Noël

**Affiliations:** 1 Inserm, U 844, Hôpital Saint-Eloi, Montpellier, France; 2 Université MONTPELLIER1, UFR de Médecine, Montpellier, France; 3 Service d’Immuno-Rhumatologie Thérapeutique, Hôpital Lapeyronie, Montpellier, France; University of Medicine and Dentistry of New Jersey, United States of America

## Abstract

The aim of this study was to identify new microRNAs (miRNAs) that are modulated during the differentiation of mesenchymal stem cells (MSCs) toward chondrocytes. Using large scale miRNA arrays, we compared the expression of miRNAs in MSCs (day 0) and at early time points (day 0.5 and 3) after chondrogenesis induction. Transfection of premiRNA or antagomiRNA was performed on MSCs before chondrogenesis induction and expression of miRNAs and chondrocyte markers was evaluated at different time points during differentiation by RT-qPCR. Among miRNAs that were modulated during chondrogenesis, we identified miR-574-3p as an early up-regulated miRNA. We found that miR-574-3p up-regulation is mediated via direct binding of Sox9 to its promoter region and demonstrated by reporter assay that retinoid X receptor (RXR)α is one gene specifically targeted by the miRNA. *In vitro* transfection of MSCs with premiR-574-3p resulted in the inhibition of chondrogenesis demonstrating its role during the commitment of MSCs towards chondrocytes. *In vivo*, however, both up- and down-regulation of miR-574-3p expression inhibited differentiation toward cartilage and bone in a model of heterotopic ossification. In conclusion, we demonstrated that Sox9-dependent up-regulation of miR-574-3p results in RXRα down-regulation. Manipulating miR-574-3p levels both *in vitro* and *in vivo* inhibited chondrogenesis suggesting that miR-574-3p might be required for chondrocyte lineage maintenance but also that of MSC multipotency.

## Introduction

Multipotent mesenchymal stromal cells or stem cells (MSCs) represent a population of adult stem cells that has a potential interest for skeletal tissue engineering, owing to their capacity to differentiate into bone, ligament, tendon or cartilage. During the embryonic process of limb formation, MSC condensation is the first step of the differentiation program toward chondrocytes that leads to the formation of cartilage tissue and ultimately, bone. This differentiation process involves complex signalling pathways that are timely and spacely regulated by several secreted factors [1]–[2]. Many of these factors are secreted by surrounding tissues and activate signaling cascades leading to proliferation and differentiation of MSCs. Among important genes, the master transcription factor Sox9 is one of the earliest markers expressed by MSCs undergoing condensation and required for the expression of cartilage-specific matrix proteins [3]. Besides the role of soluble mediators and transcription factors, growing evidence points to the role of epigenetic and microRNA (miRNA)-mediated gene control for initiating and maintaining long-term mature chondrocyte phenotype as well as controlling pathological alterations [4].

MiRNAs are small non-coding 22-nucleotide-long RNAs that participate to the post-transcriptional regulation of gene expression [5]. The importance of miRNAs on cartilage homeostasis and skeletal development was initially illustrated by experiments with cartilage specific *Dicer*-null mice [6]. *Dicer* deficiency resulted in reduced proliferation of chondrocytes in conjunction with enhanced differentiation into post-mitotic hypertrophic chondrocytes. Since then, a small number of miRNAs have been reported to be involved in modulating MSC differentiation or their expression altered in pathological situations such as osteoarthritis or rheumatoid arthritis [7]. Some miRNAs, such as miR-23b, -337, -365, positively regulate MSC differentiation [8]–[10] while others, miR-199a, -194, -455, work as repressors of chondrogenesis [11]–[13].

In the present study, we screened for miRNAs that can be modulated during the differentiation of MSCs toward chondrocytes. We identified a novel miRNA, miR-574-3p, whose expression was increased during chondrogenesis. By modulating its expression, we revealed that miR-574-3p inhibits the differentiation of hMSCs towards chondrocytes, suggesting a possible negative feedback loop. Retinoid X Receptor (RXRα) was identified as a direct target of miR-574-3p and an important regulator of MSC chondrogenesis. Our findings support a previously uncharacterized function for miR-574-3p as an inhibitor of differentiation and suggest that a threshold level of RXRα is required for adult MSC chondrogenesis and cartilage formation.

## Materials and Methods

### Cell Culture

Primary human MSCs were isolated from patients after written informed consent and approval by the General Direction for Research and Innovation, the Ethics Committee from the French Ministry of Higher Education and Research (registration number: DC-2009-1052). MSCs were expanded and characterized as previously described [14]–[15]. For chondrogenic differentiation, MSCs were cultured in pellets (2.5×10^5^ cells/500 µl) in DMEM medium with 1 mM sodium pyruvate, 170 µM ascorbic-2-phosphate acid, 350 µM proline, 1X ITS, 100 nM dexamethasone (Sigma, Le Pont-de-Claix, France), 100U penicillin/streptomycin (P/S) and 10 ng/ml TGF-β3 (R&D Systems, Lille, France) (chondrogenic medium) or, same medium without TGF-β3 for control conditions (control medium). Adipogenic differentiation was induced by culture of MSCs (8×10^3^ cells/cm^2^) in DMEM/F-12 medium containing 5% fetal calf serum (FCS), 100U P/S, 16 µM biotin, 18 µM panthotenic acid, 100 µM ascorbic acid, 1 µM dexamethasone, 60 µM indomethacin, 450 µM IBMX and 10^−6^ M rosiglitazone (Sigma). Osteogenic differentiation was obtained after culture of MSCs (3×10^3^ cells/cm^2^) in DMEM medium containing 10% FCS, 100U P/S, 2 mM L-glutamine, 50 µg/ml ascorbic acid and 100 nM dexamethasone. C-20A/4 chondrocytes were cultured in DMEM medium supplemented with 10% FCS, 2 mM L-glutamine and 100U P/S.

### MicroRNA Array Analysis

Total RNA was extracted from MSCs and MSC-derived chondrocytes (12 h; day 3) using miRNeasy kit (Qiagen S.A., Courtaboeuf, France). MiRNA expression profiling was performed by the MGX IRB Affymetrix platform using Affymetrix microarrays and sequences were obtained from the Sanger miRBase, release 11.0. Labeling and hybridization were performed according to manufacturer’s protocol with 800 ng RNA. Raw data were normalized and additional data analysis was performed as described previously [16]. All data comply with the MIAME guidelines and the complete dataset has been deposited in the Gene Expression Omnibus (GEO) online database under the accession number E-MTAB-1544. MiRNA level fold change (FC) at 12 h or day 3 versus day 0 was calculated and results are expressed as the ratio of miRNA in differentiation conditions on control conditions.

### Isolation of RNA and Quantitative RT-PCR

Total RNA, including miRNAs, was extracted using the miRNeasy kit (Qiagen). For miRNA amplification, RNA was polyadenylated using E. Coli poly(A) polymerase (NEB, Evry, France), reverse-transcribed with a poly(T) adapter and used in SYBR Green PCR kit (Applied Biosystem Meylan, France). The miRNA-specific forward primer (5′-CACGCTCATGCACACACCCACA) and the reverse primer complementary to the poly(T) adapter were used as described [17]. For mRNA quantification, PCR was done with the SYBR Green PCR kit, using specific primers (Table S1). The relative abundance of miRNAs or mRNAs was normalized to the expression of RPS9 mRNA and calculated using the ΔΔCt method [18].

### Plasmid Construction and Dual-luciferase Reporter Assays

RXRα 3′UTR was amplified using the Platinum® *Taq* DNA Polymerase (Life Technologies SAS, Saint Aubin, France) and cloned into the psiCHECK™ reporter plasmid (Promega, Charbonnières, France) using primers carrying restriction sites for XhoI (forward: 5′-CCGCTCGAGGAGGGCTGGGACTGTTTCGT) and NotI (reverse: 5′-ATAAGAATGCGGCCGCAACGAACTGAATGGCGATGT). Mutated RXRα 3′UTR was obtained by mutating the miR-574-3p seed sequence (5′-TGGAAAGTGTGAGAGGAGAAACAAAATCGTCTATGTTAAAATACATCGCCATTCAGTTCGTT), using the QuickChange® Site-Directed Mutagenesis Kit (Stratagene, Massy, France). Luciferase activity was assessed after transfection of C20A4 cells using the Dual Luciferase® Reporter Assay System (Promega) and expressed as the mean ratio of *Firefly* luciferase to *Renilla* luciferase activity.

### Cell Transfection

MSCs were transfected with 50 nM of premiR or antagomiR oligonucleotides using Oligofectamine™ (Life Technologies SAS), on days −4 and −1 before inducing their differentiation. Chondrocytes were transfected using Lipofectamine™ reagent (Life Technologies SAS) and cultured for 48 h before recovery.

### Chromatin Immunoprecipitation

C-20/A4 cells were transfected with pcDNA3-Sox9 vectors [19] for 48 h and fixed with 1% paraformaldehyde. Nuclei were lysed using RIPA buffer (10 mM Tris-HCl pH8, 1 mM EDTA, 500 µM EGTA, 140 mM NaCl, 1% triton X100, 0.1% Na-deoxycholate, 0.1% SDS, protease inhibitor cocktail (Sigma)) followed by sonication. Chromatin was immunoprecipitated with 2 µg of antibodies and 25 µl of protein A coupled agarose beads at 4°C, overnight and, digested with 20 µg/ml of proteinase K. Immunoprecipitated DNA (1/100) was used as template for RT-qPCR using sets of primers (Table S1).

### Western Blot

Proteins (20 µg) were loaded on a 10% poly-acrylamide gel and allowed to migrate before transfer onto a PVDF membrane. Membranes were incubated with anti-SOX9 (1∶1000) (Cell Signaling, Ozyme, Saint-Quentin en Yvelines, France) or anti-β-actin (Sigma) antibodies in 5% milk, 0.1% Tween 20 at 4°C, overnight. Proteins were subsequently visualized using horseradish peroxidase-linked secondary antibodies and enhanced chemiluminescence.

### Ectopic in vivo Bone Formation

SCID mice aged 9–10 weeks were grown in our animal facility. The protocol was approved by the Committee on the Ethics of Animal Experiments in Languedoc-Roussillon (CEEA-LR 36) (Permit Number: CEEA-LR-1042). hMSCs transfected with premiR or antagomiR oligonucleotides were mixed with BMP-2-expressing C3H10T1/2 cells (ratio 1∶10 respectively) and 5×10^4^ cells were implanted in the *Tibialis anterior* muscle as described [20]. Mice were euthanatized 15 days later and hind paws fixed in 4% paraformaldehyde for 7 days. Limbs were analyzed by Micro Computed Tomography (µCT) using SkyScan (Bruker, Kontich, Belgium). The 3D reconstruction of X-ray imaging and bone volume measurements were performed using Avizo® software (VSG, Merignac, France). Histological sections from muscles after paraffin embedding were stained with Safranin O and Fast Green.

### Statistical Analysis

Statistical analysis was performed using GraphPad Software (San Diego, CA). Values are given as mean ± SEM of separate experiments. Comparison between several groups used one-way ANOVA followed by Dunnett *post hoc* test or a Student’s *t* test for two groups. Differences were considered significant when p<0.05.

## Results

### miR-574-3p is up-regulated during the Early Phases of MSC Differentiation towards Chondrocytes

**Figure 1:**
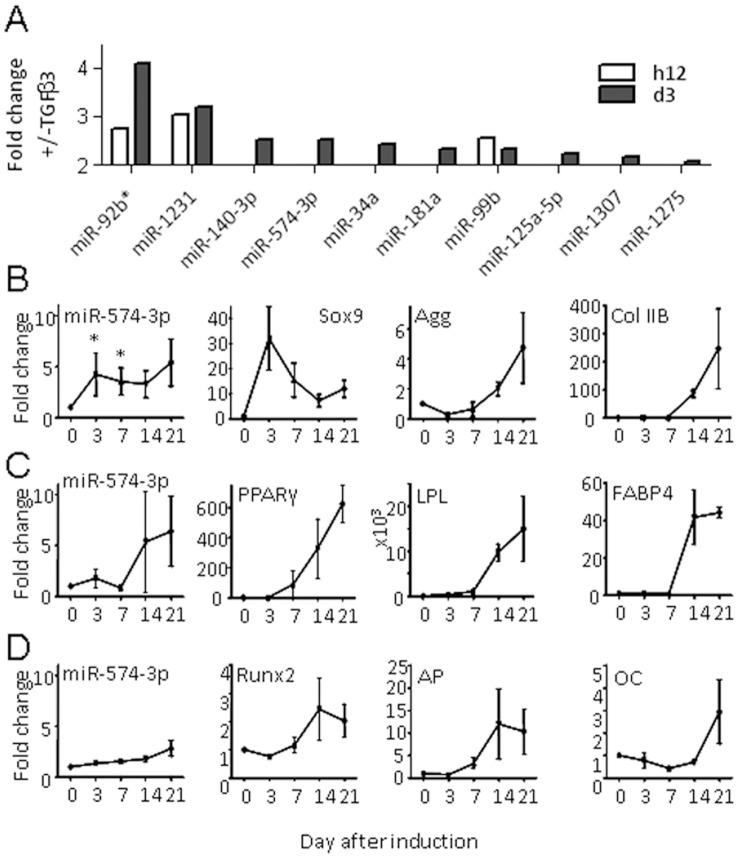
Expression of miRNAs during differentiation of MSCs. (A) Up-regulated miRNAs at 12 h and day 3 after chondrogenesis induction. (B-D) Expression of miR-574-3p and specific markers during the differentiation of MSCs towards chondrocytes (B), adipocytes (C) or osteoblasts (D). Agg: Aggrecan, Col IIB: collagen type II variant B; PPARγ: peroxisome proliferator-activated receptor, LPL: lipoprotein lipase; FABP4: fatty acid binding protein 4; Runx2, OC: osteocalcin; AP: alkaline phosphatase. Results are expressed as fold change relative to day 0 (n = 3); * *P*<0.05.

The objective of the present study was to identify miRNAs that are specifically regulated during the chondrogenic differentiation of MSCs and eliminating those induced in response to 3D environment or aggregate-induced hypoxia. We therefore compared the expression of miRNAs in MSCs cultured in pellets, either induced to differentiate in presence of the chondro-inducer TGF-β3 (differentiation medium) or cultured in absence of growth factor (control medium), for 12 h and 3 days. Expression of miRNAs in MSCs at each time point was normalized to their expression at day 0 and then data were presented as the ratio of fold changes in MSCs cultured with TGF-β3 on MSCs cultured without TGF-β3. Using a threshold of 2, we found that expression of few miRNAs was modulated after 12 h while 2 and 10 miRNAs were down- and up-regulated, respectively, at day 3 (Fig. 1A). Of these, miR-140 which has been previously shown to be increased during the differentiation of MSCs to chondrocytes, displayed the same range of modulation and expression profile than miR-574-3p. We therefore decided to focus our attention on miR-574-3p which was among the fourth most up-regulated miRNAs [21]. We first determined the expression profile of miR-574-3p in MSCs undergoing differentiation towards chondrocytes, osteoblasts or adipocytes. Using RT-qPCR, we confirmed that expression of miR-574-3p increased by a four-fold factor at day 3 of chondrogenesis and up-regulation maintained for the 3 weeks of differentiation (Fig. 1B). On the contrary, expression of miR-574-3p did not significantly enhance during adipogenesis (Fig. 1C) or osteogenesis (Fig. 1D). However, miR-574-3p levels tended to increase at late stages of adipogenic and osteogenic differentiation (days 14 and 21) (Fig. 1C, D). Although up-regulation of miR-574-3p tended to occur in late adipocytes, increased expression of miR-574-3p was primarily associated with the early commitment of MSCs towards chondrocytes.

### miR-574-3p Targets RXRα

**Figure 2:**
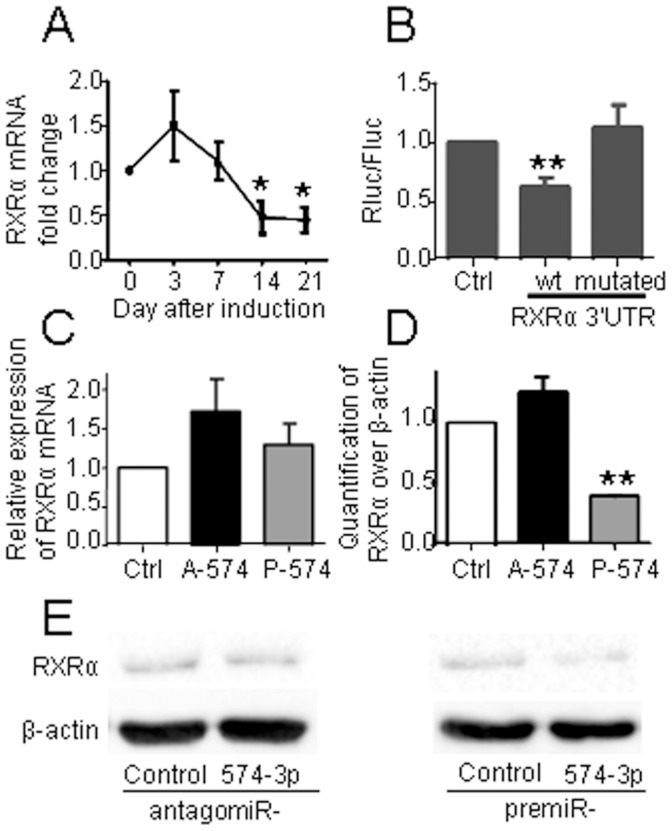
RXRα is a target of miR-574-3p. (A) RXRα expression during TGF-β3 induced chondrogenesis of hMSC. (B) C-20/A4 chondrocytes were transfected with premiR-574-3p oligonucleotides and psiCHECK-2 luciferase reporter plasmid carrying null (Ctrl: control), wild type (wt) or mutated RXRα 3′UTR sequences. Results are expressed as the ratio of Renilla (Rluc) over Firefly luciferase (Fluc) unit normalized to control plasmid (n = 6). (C) Relative expression of RXRα mRNA after transfection of C-20/A4 cells with the average premiR- plus antagomiR-control (Ctrl), premiR-574-3p (P-574) or antagomiR-574-3p (A-574) (n = 3). (D) Quantification of band intensities for RXRα protein normalized to β-actin protein after transfection of premiR- or antagomiR-control, premiR- or antagomiR-574-3p and detected by western blot as shown in (E) (n = 3). (E) Representative western blots with anti-RXRα and anti-β-actin antibodies. *p<0.05.

Because the expression of miR-574-3p increased during chondrogenesis, we hypothesized that the targeted protein may be an inhibitor of differentiation. We thus searched for potential targets of miR-574-3p using the TargetScan prediction algorithm. Among the list of putative targets, we selected RXRα which is a known inhibitor of chondrogenesis [22]. By RT-qPCR, we quantified the expression of RXRα during chondrogenesis and observed a stable expression for the first week and then a significant decrease of RXRα mRNA by day 14 and 21 (Fig. 2A). To firmly demonstrate that RXRα is a target of miR-574-3p, we cloned part of the wild-type 3′UTR sequence of RXRα, containing the seed sequence for miR-574-3p, in a luciferase reporter gene. We found that miR-574-3p ectopic expression significantly reduced the luciferase activity of the plasmid containing the 3′UTR of RXRα as compared to the control plasmid confirming the functional binding of miR-574-3p on its target (Fig. 2B). Moreover, mutation of 4 nucleotides in the seed sequence of RXRα 3′UTR totally abolished this effect (Fig. 2B). Finally, we showed that transfection of premiR or antagomiR oligonucleotides had poor effect on the mRNA expression levels of RXRα (Fig. 2C). On the contrary, down-regulation of miR-574-3p using an antagomiR approach tended to enhance the protein level of RXRα while the premiR approach significantly down-regulated the RXRα protein (Fig. 2D-E). Altogether, these results clearly demonstrated that RXRα is one specific target of miR-574-3p.

### miR-574-3p is Positively Modulated by Sox9

**Figure 3:**
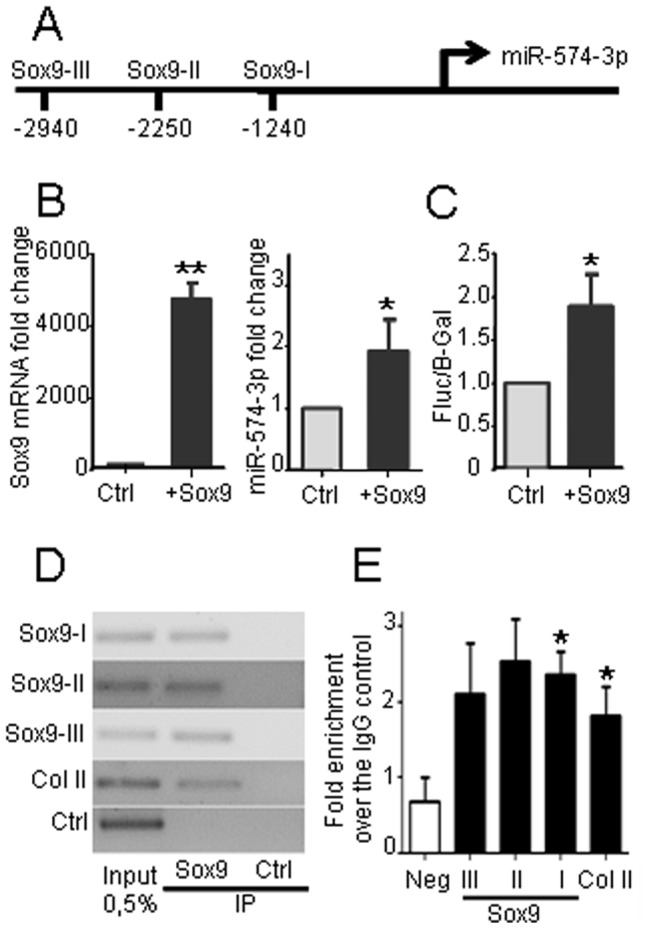
Transcription of miR-574-3p is regulated by Sox9. (A) Scheme of the three kilobases region upstream of miR-574-3p start codon. (B) Expression of Sox9 mRNA (left panel) and miR-574-3p mRNA (right panel) after transfection of C-20/A4 chondrocytes with Sox9 or control (Ctrl) plasmid (n = 3). (C) Regulation of miR-574-3p promoter. A 2 kb sequence of miR-574 promoter was cloned into a luciferase reporter system and Firefly luciferase activity (Fluc) was normalized over β-galactosidase (β-Gal) control plasmid (n = 3). (D) Representative ChIP experiment in C-20/A4 cells. Chromatin was immunoprecipitated with specific anti-Sox9 or isotype control (Ctrl) antibodies and genomic DNA fragments were amplified using specific primers by PCR. Collagen II (col II) is used as a control target gene of Sox9. (E) qPCR analysis of DNAs from panel (D) as expressed as fold enrichment over negative control using the formula (2^[(Ct input-Ct IP)-(Ct input-Ct Ctrl)]^) (n = 3). * p<0.05.

In order to determine whether the up-regulation of miR-574-3p at early stages of differentiation is transcriptionally dependent on Sox9, the master gene of chondrogenesis, we analyzed the promoter region of miR-574-3p using the MIR@nt@n software [23]. Three putative binding sites for Sox9 were identified in the promoter sequence suggesting that Sox9 may modulate miR-574-3p expression (Fig. 3A). After Sox9 transfection in the C-20/A4 chondrocyte line, the expression levels of Sox9 and miR-574-3p are concomitantly and significantly increased (Fig. 3B). The regulation of miR-574-3p by Sox9 was further investigated using a reporter plasmid containing the Firefly luciferase gene under control of the 2 kb sequence upstream of the start codon for miR-574-3p. Transfection of Sox9 positively regulated the luciferase activity (Fig. 3C), to a level similar to the RNA up-regulation (Fig. 3B). Finally, to confirm the binding of Sox9 to the miR-574-3p promoter, we performed ChIP experiments using anti-Sox9 antibody. Sox9 bound to the three binding sites in the promoter sequence of miR-574-3p and to the promoter sequence of collagen type II which is a direct target of Sox9 (Fig. 3D-E). Altogether, these results demonstrated that Sox9 binds to the promoter region of miR-574-3p and positively regulates its transcription during chondrogenesis.

### Over-expression of miR-574-3p Inhibits *in vitro* Chondrogenesis

**Figure 4:**
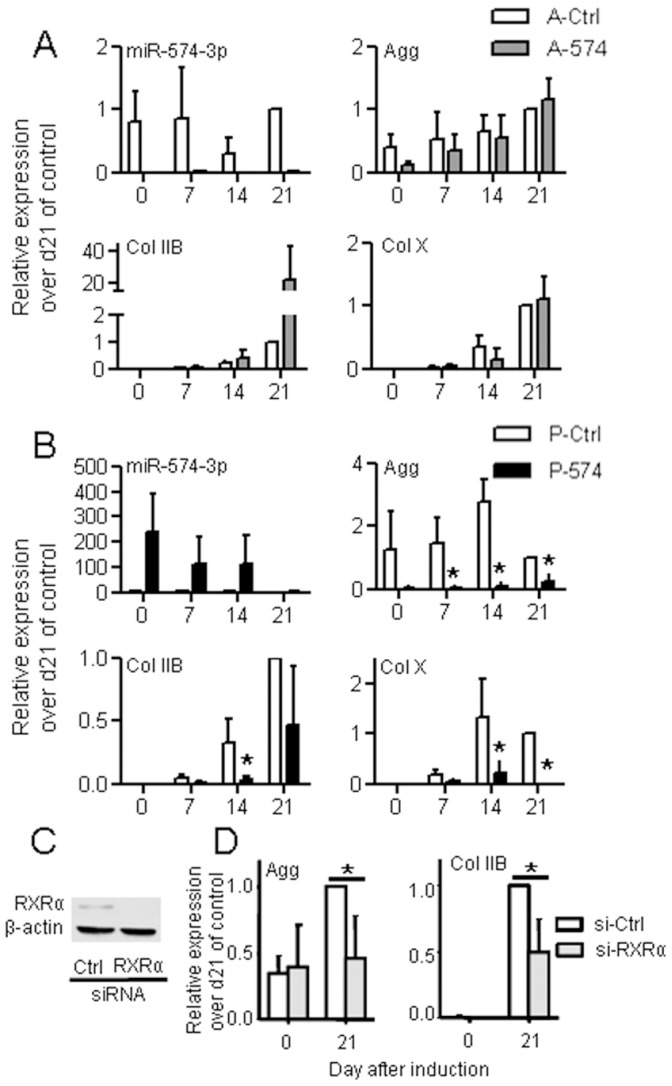
Expression of miR-574-3p and chondrocyte markers at different time points during the chondrogenic differentiation of hMSCs. (A) MSCs were transfected with antagomiR-control (A-Ctrl) or antagomiR-574-3p (A-574). (B) MSCs were transfected with premiR-control (P-Ctrl) or premiR-574-3p (P-574) (C) Expression of RXRα protein in MSCs transfected with control (si-Ctrl) or anti-RXRα siRNA (si-RXRα) and detected by Western blot at 48 h, (D) MSCs were transfected with control (si-Ctrl) or anti-RXRα siRNA (si-RXRα). Results are expressed as gene expression relative to expression of the control at day 21 (N = 3). Agg: aggrecan, Col IIB: collagen type II variant B, Col X: collagen type X; *p<0.05.

The above results indicated that Sox9 can up-regulate miR-574-3p, which in turn reduce the expression levels of RXRα. RXRα was previously shown to inhibit chondrogenesis through direct binding to retinoid X receptor elements (RXRE) in the promoter of Sox9 [22]. Indeed, decreasing miR-574-3p levels in MSCs should result in up-regulation of RXRα and chondrogenesis inhibition. To test this hypothesis, we investigated the *in vitro* effect of synthetic premiR or antagomiR oligonucleotides complementary to miR-574-3p on MSCs induced to differentiate in presence of TGF-β3. Although a high down-regulation of miR-574-3p was observed after antagomiR transfection, we could not observe significant regulation of chondrogenic markers (Fig. 4A). Contrary to our hypothesis, the strong up-regulation of miR-574-3p using the premiR approach resulted in the inhibition of the chondrocytic markers, aggrecan, collagen type II variant B and collagen type X (Fig. 4B). While the expression of collagen type II variant B was significantly reduced at day 14, it tended to decrease by day 21; a time point where miR-574-3p was no more over-expressed. Of importance, the decreased expression of aggrecan was significant from day 7 to 21 and that of collagen X from day 14 to 21 (Fig. 4B). To further investigate the role of RXRα in the present cell differentiation model and mimic the effect of premiR-574-3p, we decided to transfect a siRNA specific for RXRα in MSCs before inducing their differentiation. After transfection, a strong inhibition of RXRα protein level was observed by western blotting (Fig. 4C). Similar to results obtained with premiR-574-3p, we observed a significant down-regulation of both aggrecan and collagen type II variant B (Fig. 4D). Thus, over-expressing small interfering RNAs (premiR-574-3p or anti-RXRα siRNA) in MSCs resulted in an equivalent inhibition of chondrogenic differentiation suggesting that RXRα expression is required at the onset of chondrogenesis.

### In vivo Modulation of miR-574-3p Inhibits Endochondral Bone Formation

**Figure 5:**
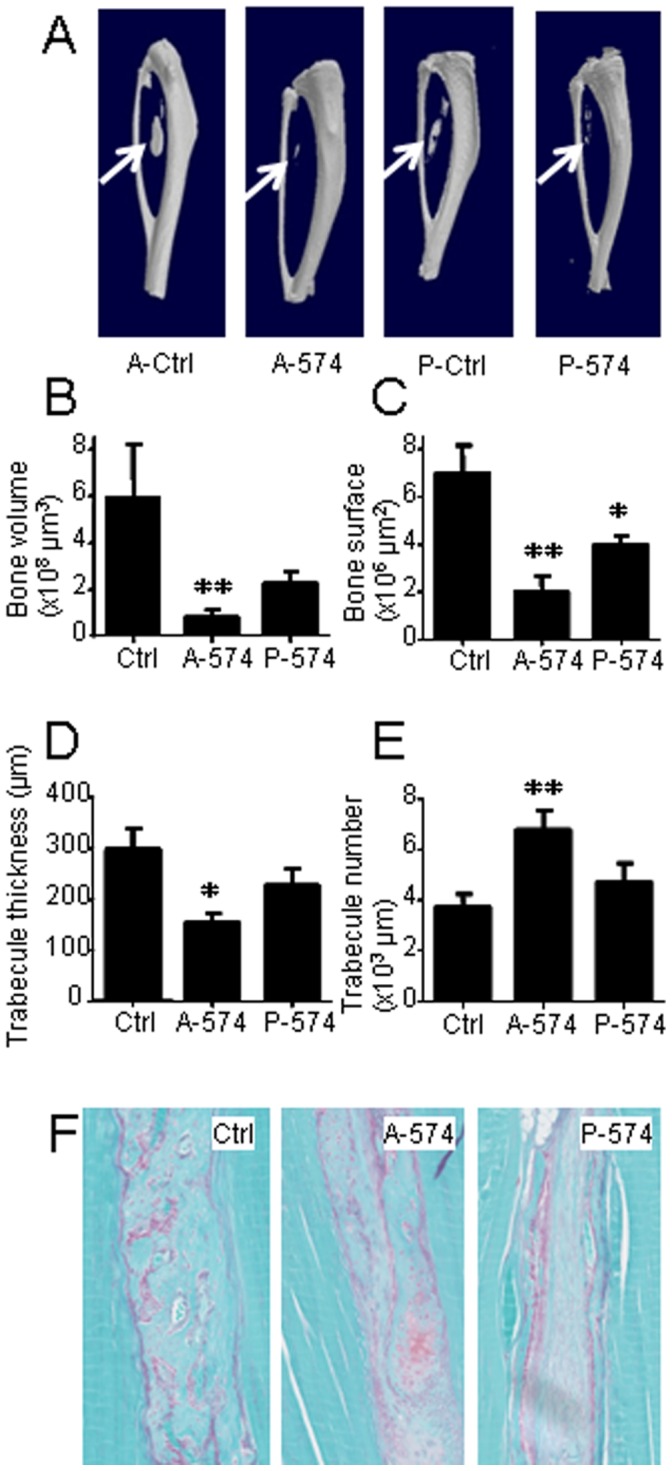
In vivo endochondral ossification after co-implantation of BMP2-expressing C3H10T1/2 MSCs and hMSCs in *Tibialis anterior* muscle of SCID mice. hMSCs were previously transfected with premiR- or antagomiR-control (Ctrl), premiR-574-3p (P-574) or antagomiR-574-3p (A-574) oligonucleotides. (A) Representative micro-CT images of neotissue formation. (B–E) Quantitative analysis of the indicated bone parameters of the neotissues as shown in (A) (n = 4 mice per group; Ctrl is the average of data from premiR-Ctrl and antagomiR-Ctrl). (F) Representative histological sections of the neotissues stained with Safranin O and fast Green (magnification ×10). *p<0.05.

To confirm the *in vitro* experiments, and in absence of an easily available model of *in vivo* cartilage formation which might provide reliable and quantitative assessment of parameters specific for cartilage, we used a model of endochondral ossification. In this model, BMP-2-expressing murine MSCs can differentiate into chondrocytes and form cartilage which is progressively replaced by bone [20]. Neobone formation can be monitored by µCT imaging and bone parameters accurately recorded. The interest of using this model in the present study is that premiR- or antagomiR-transfected hMSCs may be combined with one-tenth of murine MSCs expressing BMP-2 which will induce the differentiation of the hMSCs. Indeed, hMSCs transfected with antagomiR-574-3p or premiR-574-3p oligonucleotides were injected with BMP-2-expressing murine MSCs in the *Tibialis anterior* muscles of SCID mice. Smaller ectopic mineralized tissue masses were detected at the operated site near the tibia when cells expressed both premiR-574-3p and antagomiR-574-3p as compared to control premiR or antagomiR (Fig. 5A). When antagomiR-574-3p was expressed in hMSCs, analysis of bone parameters revealed a significant decrease of bone volume, bone surface, trabecular thickness, bone density (not shown) and increase of trabecular number (Fig. 5B-E). Histologically, the new formed tissues were mainly composed of bone in mice receiving MSCs expressing control oligonucleotides whereas a fibrous tissue and some infiltration of immune cells were seen in neotissues expressing antagomiR-574-3p (Fig. 5F). Using the complementary approach where MSCs expressed premiR-574-3p, we observed that, except for the bone surface which was significantly reduced, most of the bone parameters tended to decrease but were not significantly altered as compared to controls (Fig. 5B-F). Indeed, increased expression levels of miR-574-3p, and likely the resulting down-regulation of RXRα levels, slightly impair cartilage formation and the resulting heterotopic ossification. Surprisingly, stronger inhibition was observed with the antagomiR approach. Whether this effect was due to the *in vivo* regulation of other target genes or the BMP-dependent ossification model still remains to be understood.

## Discussion

The aim of this study was to identify new miRNAs regulating the chondrogenic differentiation of MSCs. Using large scale miRNA analysis, we identified that miR-574-3p was among the four most up-regulated miRNAs at day 3 of chondrogenesis. The most up-regulated miRNAs were miR-92b which is involved in proliferation and cancer [24]–[25] and miR-1231 whose function has only recently been reported [26]. Because miR-574-3p exhibited the same kinetics of expression as miR-140 whose role on chondrocyte phenotype has been already reported [21]–[27] and because no publications on miR-574-3p in MSCs or chondrocytes were available at the time of identification, we decided to investigate in more details the role of this miRNA during the differentiation of MSCs.

We showed here that miR-574-3p expression increased at the early stages of chondrogenesis and maintained elevated throughout differentiation. The early induction of miR-574-3p expression was not observed during osteogenesis or adipogenesis and was directly dependent on Sox9, the master gene of chondrogenesis. This up-regulation of miR-574-3p suggested that it probably target genes that are inhibitors of differentiation. Using prediction software, we selected RXRα as being among possible targets with the highest prediction rates, being highly conserved across species and already described as an inhibitor of differentiation [22]. We here validated RXRα as an effective direct target of miR-574-3p and reported that its expression progressively decreased during the differentiation process. These results are therefore in accordance with previous studies reporting that over-expression of RXRα inhibited Sox9 reporter activity in primary mouse limb mesenchymal cultures while the dominant negative form potently activates this reporter [22]. The authors also reported that increase in cartilage formation was blocked by a histone deacetylase (HDAC) inhibitor, indicating that recruitment of HDACs is essential for chondrocyte differentiation. Indeed, decrease of retinoic acid (RA) in chondroprogenitor cells would allow HDAC recruitment to RARs and promote differentiation [28]. A more recent study showed that MSC treatment with All-Trans-Retinoic Acid (ATRA) inhibits chondrogenesis, by negatively regulating TGF-β pathway [29]. On the contrary, RXRα was reported to act as a differentiation-inducing cofactor in other cell systems where it may interact with vitamin D receptor (VDR) to regulate osteogenesis and matrix calcification, but also with peroxisome proliferator-activated receptor (PPAR)-γ to induce MSC adipogenesis upon PPAR-γ ligand binding [30].

The present study added arguments to the compelling evidence for a requirement for RXR- and RAR-mediated repression during the chondrogenic differentiation of murine mesenchymal progenitor cells [31]. Indeed, we report a significant down-regulation of RXRα expression during the last stages of MSC differentiation. Our work further suggests that miR-574-3p may participate to a regulatory loop. Since RXRα was previously demonstrated to inhibit Sox9 activity, RXRα has to be down-regulated for chondrogenic differentiation to occur; this may be achieved via Sox9-mediated up-regulation of miR-574-3p.

While we demonstrated that miR-574 may regulate chondrogenesis through the regulation of RXRα, we cannot exclude that it acts through the inhibition of one or several other target mRNAs. Indeed, cullin 2 (CUL2) and mesoderm development candidate 1 (MESDC1) were recently described as being targets of miR-574-3p in gastric and bladder cancer cells, respectively [32]–[33]. In those cancer cells, miR-574-3p had tumor suppressor activity. Consistent with these studies, miR-574-3p has also been shown to negatively regulate the proliferation of keratinocytes by targeting p63, a homolog of p53 which plays a crucial role in epithelial development [34]. Loss of p63 resulted in a complete absence of proliferating cells in the epidermis and accelerated differentiation. Although shown in other models, these recent data further support the role of miR-574-3p as a regulator of differentiation.

In spite of the apparent regulatory effect of miR-574-3p on RXRα based on the its expression during chondrogenesis, we obtained unexpected results using premiR and antagomiR expression both *in vitro* and using the *in vivo* model of heterotopic ossification. The diminution of RXRα level in MSCs, before differentiation induction, resulted in chondrogenesis inhibition. This was clearly observed using both a premiR- and a siRNA-mediated approach suggesting that threshold levels of RXRα are required for initiating differentiation of MSCs. Although we cannot exclude that expression of several target genes, others than RXRα, may be modulated by this approach, we observed a distinct drop in RXRα protein before inducing the differentiation process. It has been suggested elsewhere that the impact of an altered balance of RXR and RAR might have severe effects and alter the entire RXRα signaling cascades. As an example, in cancer cells, RXR homodimerization leads to p21 up-regulation, induction of cell cycle arrest and apoptosis of the cells whereas presence of RAR interferes with RXR ligand-mediated p21 induction [35]. These data suggest that the level of RXR ligand-mediated growth inhibitory effect could be manipulated by the overall amount of RAR and RXR. Indeed, we may speculate that low amounts of RXRα will favor the formation of heterodimers instead of homodimers and change the binding partners, leading to a different regulation of genes involved in initiation of MSC differentiation.

### Conclusions

In summary, we present evidence for a role of miR-574-3p in the Sox9-mediated regulation of MSC chondrogenesis. This result suggests that up-regulation of miR-574-3p during MSC differentiation might be required for chondrocyte lineage maintenance through the down-regulation of RXRα. Furthermore, our results show that functional inhibition of RXRα in MSCs can inhibit their differentiation towards chondrocytes. Indeed, a properly regulated level of RXRα expression seems to be required at the initiation of cartilage formation. This finding indicates that RXRα might be involved in the maintenance of MSC multipotency and stem cell state. Such hypothesis will be investigated in future work.

## Supporting Information

Table S1 List of oligonucleotide primers used for real-time PCR.
(DOCX)
