# Supplementary material for: Sox9-Regulated miRNA-574-3p Inhibits Chondrogenic Differentiation of Mesenchymal Stem Cells
Source: PLoS One. 2013 Apr 23;8(4):e62582. doi: 10.1371/journal.pone.0062582 (PMC3633883; doi:10.1371/journal.pone.0062582)
Supplement: Table S1 — List of oligonucleotide primers used for real-time PCR. (DOCX) [file pone.0062582.s001.docx]

Table S1: List of oligonucleotide primers used for real-time PCR

| **Target gene** | **Primers (forward and reverse)** |
| --- | --- |
| **RPS9** | AAGGCCGCCCGGGAACTGCTGAC  ACCACCTGCTTGCGGACCCTGATA |
| **Collagen II variant B** | CAGACGCTGGTGCTGCT  TCCTGGTTGCCGGACAT |
| **Collagen X** | TGCTGCCACAAATACCCTTT  GTGGACCAGGAGTACCTTGC |
| **Aggrecan** | ATGCCCAAGACTACCAGTGG  TCCTGGAAGCTCTTCTCAGT |
| **Sox9** | AGGTGCTCAAAGGCTACGAC  GTAATCCGGGTGGTCCTTCT |
| **RXRα** | ACATTTCCTGCCGCTCGATT  ATGTCCTCGCTGCTGCTGAC |
| **PPAR-γ** | GTGGCCGCAGAAATGACC  CCACGGAGCTGATCCCAA |
| **LPL** | GTCCGTGGCTACCTGTCATT  TGGATCGAGGCCAGTAATTC |
| **FABP4** | AGTGAAAACTTTGATGATTATATG  CCATGCCAGCCACTTTCCT |
| **Runx2** | GATCCCCCCAAGAAGGCACAGACAGATTCAA  AGCTTAAAAACCAAGAAGGCACAGACAGATC |
| **AP** | GACAGACACACCATGAGAACC  CTAGCTCGTCACAGTCAGGG |
| **OC** | CACTCCTCGCCCTATTGGCC  GCCAACTCGTCACAGTCCGG |
| **RNU6** | AATTCGTGAAGCGTTCCATATTTT  GCGAGCACAGAATTAATACGACT |
| **(ChIP) Collagen 2a1** | ACCTGTGAATCGGGCTCTGT  CCCACTGGACCTCGTCTCTC |
| **(ChIP) Sox9-I** | CGTCAACATCTCGCCATTTTT  GCCCTAGTCTCTCCCTTTCAA |
| **(ChIP) Sox9-II** | AAGGGACTGTGCCGAGCAAT  TGAGAGTGGGTGGAAACTGCTT |
| **(ChIP) Sox9-III** | TCATCACACCACTGCACTCC  TGAAACCCTATCCCTTCCATT |
| **(ChIP) Negative** | TTCAAAGGTGGCAATTGTAGCA CAAAGTCTTCATAGGAGGGCATGA |
